# Supplementary material for: The Effect of Protein Supplementation versus Carbohydrate Supplementation on Muscle Damage Markers and Soreness Following a 15-km Road Race: A Double-Blind Randomized Controlled Trial
Source: Nutrients. 2021 Mar 5;13(3):858. doi: 10.3390/nu13030858 (PMC7999032; doi:10.3390/nu13030858)
Supplement: Supplementary file 1 [file nutrients-13-00858-s001.pdf]

Supplemental table S1. Habitual dietary intake of participants in the protein and placebo group on the day of the race before and after the race, based on ITT

	Before the race			After the race		
	Protein *n = 134*	Placebo *n = 127*	P-value	Protein *n = 134*	Placebo *n = 127*	P-value
Energy intake, kcal	853 ± 391	854 ± 349	0.98	1425 ± 601	1423 ± 621	0.97
Protein intake, g	28.9 ± 15.2	29.7 ± 14.2	0.66	47.2 ± 27.5	49.9 ± 31.9	0.46
Protein intake, g/kg/d	0.40 ± 0.20[a]	0.43 ± 0.19[b]	0.34	0.65 ± 0.33[c]	0.67 ± 0.34[d]	0.69
Animal protein, %	39.6 ± 23.9	40.8 ± 24.6	0.67	52.7 ± 21.4	52.0 ± 24.9	0.82
Plant protein, %	60.5 ± 24.0	59.1 ± 24.5	0.65	47.3 ± 21.5	48.1 ± 25.0	0.79
Protein, en%	14.2 ± 5.5	14.1 ± 4.4	0.90	13.6 ± 5.9	14.5 ± 6.6	0.26
Fat intake, en%	25.5 ± 11.7	28.2 ± 11.2	0.057	33.5 ± 11.3	33.6 ± 13.4	0.98
Carbohydrate intake, en%	57.0 ± 12.5	54.8 ± 12.3	0.16	43.9 ± 12.3	43.6 ± 14.2	0.83
Alcohol intake, g	0.0 (0.0 – 0.0)	0.0 (0.0 – 0.0)	0.98 ‡	7.9 (0.0 – 25.9)	0.0 (0.0 – 18.9)	0.25 ‡
Non-drinker, n (%)	-	-	-	61 (50)	67 (57)	0.29
Low- drinker, n (%)	-	-	-	31 (25)	31 (26)	
Moderate drinker, n (%)	-	-	-	27 (22)	14 (12)	
High drinker, n (%)	-	-	-	3 (3)	6 (5)	

Data are presented as mean ± SD or median (interquartile range (IQR)). en%, energy percentage; ITT, Intention-to-treat analysis.

[a] *n* = 97, [b] *n* = 106, [c] *n* = 97, [d] *n* = 106.

P-values for differences between groups were derived by independent samples t-test unless indicated otherwise. ‡ Derived by Mann-Whitney U-test.

Supplemental table S2. Baseline and post-race NPRS scores for perceived **lower extremity** muscle soreness, based on PP

		**Protein**	**Placebo**	**P-value**
**Time**				
		n = 150	*n = 150*	
Baseline	Worst muscle soreness	0.67 ± 1.61	0.66 ± 1.58	0.93
	Least muscle soreness	0.27 ± 0.78	0.20 ± 0.69	0.53
	Average muscle soreness past 24 h	0.45 ± 1.08	0.44 ± 1.14	0.96
		n = 137	*n = 139*	
1 day post-race	Worst muscle soreness	4.02 ± 2.81	3.60 ± 3.00	0.21
	Least muscle soreness	1.77 ± 1.97	1.27 ± 1.74	**0.017**
	Average muscle soreness past 24 h	2.96 ± 2.27	2.46 ± 2.38	**0.039**
		n = 124	*n = 127*	
2 days post-race	Worst muscle soreness	2.71 ± 2.35	2.38 ± 2.62	0.15
	Least muscle soreness	1.18 ± 1.54	1.13 ± 1.66	0.30
	Average muscle soreness past 24 h	1.93 ± 1.87	1.68 ± 2.08	0.09
		n = 105	*n = 109*	
3 days post-race	Worst muscle soreness	1.36 ± 2.02	1.16 ± 1.71	0.86
	Least muscle soreness	0.50 ± 1.01	0.43 ± 0.76	0.99
	Average muscle soreness past 24 h	0.90 ± 1.48	0.79 ± 1.17	0.97

Data are presented as mean ± SD.
NPRS; numeric pain rating scale; PP, Per-protocol analysis
P-values for differences between groups were derived by Mann-Whitney U-test.

Supplemental table S3. Pressure pain thresholds with a strain gauge algometer measured 1 and 2 days post-race, based on PP

	Protein *n = 63*	Placebo *n = 69*	P-value
M. vastus lateralis, N	75.0 ± 34.6	83.2 ± 33.4	0.17
M. rectus femoris, N	80.6 ± 35.9	93.5 ± 31.3	**0.029**
M. vastus medialis, N	59.9 ± 25.9	74.9 ± 30.2	**0.003**
Average 3 muscles, N	71.8 ± 30.0	83.9 ± 27.9	**0.019**

Data are presented as mean ± SD.

M, musculus; PP, Per-protocol analysis

P-values for differences between groups were derived by independent samples t-test

Supplemental table S4. Muscle damage markers measured 1 and 2 days post-race, based on PP

	Protein *n = 63*	Placebo *n = 68*	P-value
CK, U/L	285 (172 – 516)	346 (196 – 508)	0.30
LDH, U/L	181 (144 – 201) [a]	171 (147 – 197)[b]	0.60

Data are presented as median (interquartile range (IQR)).
CK; creatine kinase, LDH, lactate dehydrogenase; PP, Per-protocol analysis, U/L, unit/liter.
[a] *n = 62*, [b] *n = 67*, due to hemolysis.
P-values for differences between groups were derived by Mann-Whitney U-test.
